# Case report of Li’s Tongbian scraping therapy for patients with severe refractory acne complicated with anxiety

**DOI:** 10.1097/MD.0000000000045052

**Published:** 2025-10-24

**Authors:** Wei-Sheng Gu, Qing-Xin Mai, Xiao-Ling Chen, Yong-Qi Liang, Yao Zhou, Fang Liu, Jun-Jun Zhang

**Affiliations:** a Department of Nursing, Shenzhen Hospital of Integrated Traditional Chinese and Western Medicine, Shenzhen City, Guangdong Province, China.

**Keywords:** acne, anxiety, Li’s Tongbian scraping therapy, nursing

## Abstract

**Rationale:**

This report presents the case of a patient with severe acne (grade IV) accompanied by moderate anxiety who was treated by Li’s Tongbian scraping therapy.

**Patient concerns:**

A 52-year-old man was suffering from recurrent acne for over 10 years, accompanied by insomnia and anxiety.

**Diagnoses:**

He was diagnosed with severe (grade IV) acne, moderate anxiety, and mild insomnia.

**Interventions:**

The patient was treated with Li’s Tongbian scraping therapy for 3 months.

**Outcomes:**

Patient’s skin inflammation had significantly subsided, his anxiety score had reduced from 63 to 43, and his insomnia severity index had decreased from 14 to 5. The patient’s overall quality-of-life had improved significantly.

**Lessons:**

Li’s Tongbian scraping therapy may serve as an optimal non-pharmacological treatment option for recalcitrant acne.

## 1. Introduction

Acne is a chronic inflammatory skin condition that affects the pilosebaceous unit. It commonly affects areas with abundant sebaceous glands, such as the face, chest, and back. Clinically, it manifests as comedones, papules, pustules, cysts, and nodules. As per epidemiological studies, acne vulgaris affects approximately 9.4% of the global population, and its incidence is rising annually.^[1]^ In China, the prevalence of acne vulgaris among college students is 41.04%.^[2]^ This condition predominantly affects the face, and 3% to 7% of all patients develop disfiguring scars. Acne can significantly impact both the physical and psychological well-being of patients, diminishing their quality-of-life.^[3]^

The currently available Western medical treatments include oral or topical retinoids and antibiotics. However, retinoids may cause adverse effects such as erythema, dryness, and skin peeling, whereas prolonged use of antibiotics can result in drug resistance. Additional therapies such as chemical exfoliation and physical modalities like laser or photodynamic therapy often entail high medical costs, which place a financial burden on the patients. These factors are frequently associated with poor treatment compliance, contributing to recurrent acne and suboptimal therapeutic outcomes.^[4]^

Li’s Tongbian scraping (also known as Gua Sha, Tongbian scraping, or Hufu copper bianstone) therapy^[5]^ is an innovative external treatment method rooted in traditional Chinese medicine (TCM). Based on the principle of “treating through unblocking and tonifying with unblocking,” it aims to promote qi and blood circulation and regulate organ function by stimulating meridians and acupoints. Scraping the skin with Li’s Tongbian stimulates the meridians on the body’s surface, effectively unblocking them. This action not only promotes qi and blood circulation but also helps to adjust the Yin and Yang balance, enhancing the functions of the internal organs. This therapy employs gentle and rhythmic techniques along with scraping oil. By doing so, it effectively opens the skin pores, enabling the smooth expulsion of pathogenic qi. These actions work in tandem to restore internal balance, thereby playing a significant role in acne treatment. This case reports a TCM nursing care approach to a patient with severe acne and moderate anxiety.

## 2. Case review

### 2.1. Patient concerns

Mr Jiang (a 52-year-old man) was suffering from acne for more than 10 years. During this time, he sought treatment at multiple hospitals in Guangzhou and Shenzhen and underwent various therapies, including topical treatments, oral antibiotics, and a combination of red and blue light therapy. Despite these efforts, his acne recurred and remained unresolved.

### 2.2. Present illness

On October 15, 2024, Mr Jiang visited a TCM nursing specialist clinic with a primary complaint of recurrent acne for over a decade. On examination, extensive lesions were noted on the face, submandibular area, scalp, and back, accompanied by localized erythema, pruritus, subcutaneous nodules, and sebum overproduction. Depressed scars were observed on facial skin.

### 2.3. Admission assessment

Skin assessment: Diagnosed with severe grade IV acne according to the Chinese Acne Treatment Guidelines (2019 revised edition).^[3]^ Psychological assessment: Scored 63 points on the self-rating anxiety scale, indicating moderate anxiety,^[6]^ and 14 points on the insomnia severity index,^[7]^ indicating mild insomnia. Accompanying symptoms: Localized burning sensation and pruritus of the skin, impairing daily activities, and sleep quality.

### 2.4. Treatment plan

**Figure 1.**
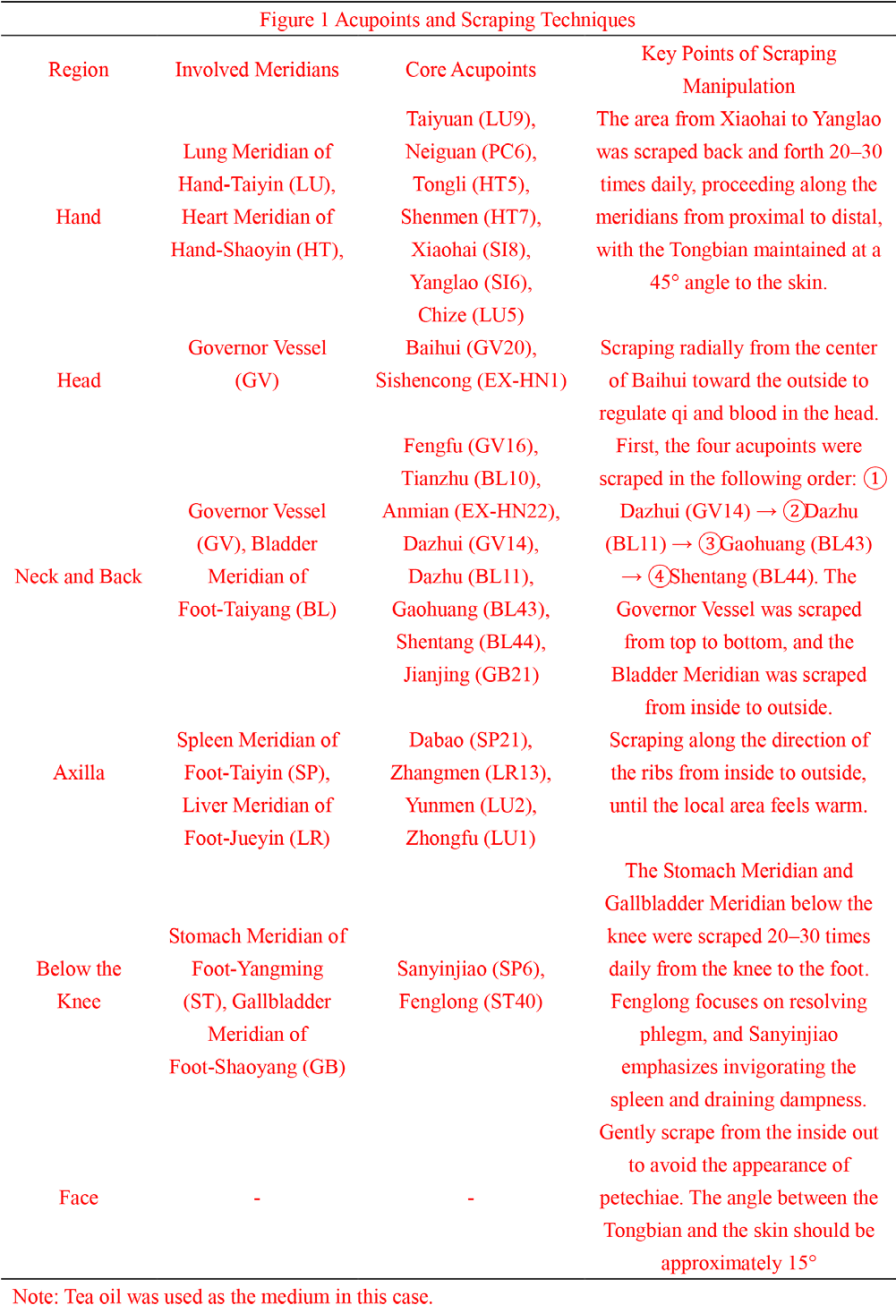
Acupoints and scraping techniques. Tea oil was used as the medium in this case.

Selection of Gua Sha acupoints and meridians^[8]^ (Fig. 1): Using Li’s Tongbian scraping therapy with brass scraping tools and scraping oil, following the principle of “slow and harmonious” operation; the treatment focused on the following meridians and acupoints.

#### 2.4.1. Hand acupoints

Taiyuan (LU9), Neiguan (PC6), Tongli (HT5), Shenmen (HT7), Xiaohai (SI8), Laoyang (SI8), and Chize (LU5) to unblock the Hand Taiyin Lung (LU) and Hand Shaoyin Heart meridians (HT).

#### 2.4.2. Head acupoints

Baihui (GV20) and Sishencong (EX-HN 1) to regulate qi and blood circulation in the head.

#### 2.4.3. Neck and back acupoints

Fengfu (GV16), Tianzhu (BL10), Anmian (EX-HN 22), Dazhui (GV14), Dazhu (BL11), Gao Huang (BL43), Shentang (BL44), and Jianjing (GB21) to relieve neck and shoulder tension and regulate qi and blood along the governor vessel (GV) and bladder meridians (BL).

#### 2.4.4. Under the axilla acupoints

Dabao (SP21), Zhangmen (LR13), Yunmen (LU2), and Zhongfu (LU1) to harmonize liver, spleen, and lung qi movement.

#### 2.4.5. Below the knee

Points along the stomach (ST) and gallbladder (GB) meridians, including the intersection of Sanyinjiao (SP6) and Fenglong (ST40) to strengthen the spleen, resolve phlegm, clear heat, and promote diuresis.

#### 2.4.6. Four wells detoxification

Scraping the tips of the hands and feet to enhance toxin metabolism.

## 3. Course of treatment

The treatment was performed once a week. Each course consisted of 3 weekly sessions, followed by a 1-week break, repeated over 3 courses. The total treatment duration was 3 months. Whole-body scraping was conducted once a week. However, daily scraping was performed from Xiaohai (SI8) to Yanglao (SI8), the points above and below the knees, respectively, 20 to 30 strokes per area. Each session lasted 30 to 40 minutes, continuing until mild skin flushing or local warmth appeared.

### 3.1. Method of operation

Back scraping: The patient sat upright while essential oil was being applied to his skin. Li’s Tongbian scraping was then performed at a 45° angle, beginning with the Dazhui (GV14), Dazhu (BL11), Gao Huang (BL43), and Shendang (BL44) points, followed by the Yang meridians (GV and BL). Scraping followed the course of the Hand Taiyin Lung (LU) and Hand Yangming Large Intestine meridians (LI). Facial scraping: The patient was asked to lie flat with a pillow under the head and keep the eyes closed. After cleansing the facial skin with a moist gauze, the patient’s face was massaged along the meridians with an appropriate amount of essential oil. After the massage, Li’s Tongbian scraping therapy board was used to apply an appropriate amount of essential oil and scrape it gently from the inside outward, along the facial muscles.

### 3.2. Precaution

The principle of “top-to-bottom, inside-to-outside” was followed, applying pressure from light to heavy within the patient’s tolerance to avoid skin damage. Facial scraping was performed gently to prevent petechiae and protect the appearance. Once the treatment was completed, wind exposure was prohibited, and the patient was allowed to bathe 4 to 6 hours later. The patient was asked to drink plenty of warm water to help expel pathogenic qi. Observation of petechiae: The skin was monitored closely. If purple-red petechiae or blisters appeared on the localized skin, the operation was immediately ceased and the patient was accordingly managed.

### 3.3. Treatment outcomes

Improvement in skin symptoms: After 3 months of treatment, most facial, submandibular, and back acne lesions had healed. Subcutaneous nodules shrank, sebum secretion decreased, and skin inflammation subsided significantly. New comedones reduced by approximately 80%, and facial scars lightened, resulting in healthier skin overall. Mental and sleep improvement: Self-rating anxiety scale scores dropped from 63 to 43 (no anxiety state), indicating that the patient had returned to a non-anxious state, with his skin condition improving significantly following his active cooperation during the treatment. The insomnia severity index scores reduced from 14 to 5, reflecting normal sleep quality (6–8 hours of continuous sleep each night and having a good mental state in the morning). At the 3-month telephonic follow-up, the patient reported no acne recurrence or related distress.

## 4. Discussion

The existing guidelines state^[3,9]^ that severe acne (grade IV), owing to its refractory nature, typically requires a combination treatment regimen, such as oral isotretinoin, topical retinoids, and antibiotics. However, the long-term use of these medications is limited owing to their adverse effects (e.g., isotretinoin can cause mucocutaneous dryness, and antibiotics can induce drug resistance), and the recurrence rate after treatment is relatively high. This issue is particularly prominent among patients with persistent inflammation or psychological stress. As a characteristic therapy derived from TCM, Li’s Tongbian Gua Sha therapy aligns with the emerging research direction of complementary treatment for acne, and it is especially suitable for patients who have failed conventional treatment or are intolerant to medications.

The guidelines emphasize that the treatment of acne requires proceeding simultaneously from multiple dimensions, such as regulating sebum secretion and inhibiting inflammatory responses.^[3]^ This principle provides a basis for comparison and reference for the multi-dimensional intervention approach of Li’s Tongbian scraping therapy in the treatment of acne, as in the present case, which not only highlights the importance of comprehensive treatment but also echoes the comprehensive intervention effect demonstrated by this therapy in the present case.

In this case, Li’s Tongbian scraping therapy achieved the dual effects of “treating the lungs and spleen and regulating the mind and body” by targeting Lung meridian points (e.g., Taiyuan and Chize) to clear heat. Heart meridian points (e.g., Shenmen and Neiguan) were selected to calm the mind. The spleen and stomach meridians, including acupoints such as Sanyinjiao and Fenglong, were stimulated to strengthen the spleen and eliminate dampness.^[5]^ Modern research has demonstrated that Tongbian scraping therapy activates the skin receptors. Through the neuroendocrine-immune network, it regulates inflammatory factors, suppresses the growth of *Propionibacterium acnes*, promotes local blood circulation, and optimizes the keratinization of hair follicle openings while enhancing sebum regulation. Moreover, mechanical stimulation from Tongbian scraping therapy activates endogenous analgesic systems, alleviating itching and burning sensations and indirectly improving sleep quality.^[5,10]^

In patients with chronic acne, moderate anxiety further exacerbates inflammatory skin responses. This case emphasized establishing trust, providing health education, and offering cognitive interventions to help the patient understand the link between emotions and acne. Gua Sha therapy also helped regulate autonomic nervous function, creating a “physical–psychological” positive feedback loop. Studies^[5,11]^ have demonstrated that anxiety can increase cortisol levels through the hypothalamic–pituitary–adrenal axis, aggravating acne.

## 5. Conclusions

This case experience preliminarily suggests that Li’s Tongbian scraping therapy may exert a certain ameliorative effect on acne patients with similar severe, long-term recurrent characteristics complicated by psychosomatic symptoms.

### 5.1. Limitations

A single case report cannot rule out the impact of individual differences on therapeutic efficacy. Rigorous randomized controlled studies are hence required to verify the efficacy and generalizability of Li’s Tongbian scraping therapy.

## Acknowledgments

The people who participated in this research and Shenzhen Hospital of Integrated Traditional Chinese and Western Medicine are appreciatively acknowledged.

## Author contributions

**Conceptualization:** Jun-Jun Zhang.

**Data curation:** Wei-Sheng Gu.

**Formal analysis:** Xiao-Ling Chen.

**Project administration:** Wei-Sheng Gu, Yong-Qi Liang, Yao Zhou, Fang Liu.

**Writing – original draft:** Qing-Xin Mai, Xiao-Ling Chen, Yong-Qi Liang.

**Writing – review & editing:** Jun-Jun Zhang.
